# Brain serotonergic activation in growth-stunted farmed salmon: adaption versus pathology

**DOI:** 10.1098/rsos.160030

**Published:** 2016-05-25

**Authors:** Marco A. Vindas, Ida B. Johansen, Ole Folkedal, Erik Höglund, Marnix Gorissen, Gert Flik, Tore S. Kristiansen, Øyvind Øverli

**Affiliations:** 1 Department of Biosciences, University of Oslo, PO Box 1041, Blindern, 0316 Oslo, Norway; 2 Uni Research AS, PO Box 7810, 5020 Bergen, Norway; 3 Bjørknes College, Oslo, Norway; 4 Institute of Marine Research, 5984 Matredal, Norway; 5 National Institute of Aquatic Resources, Section for Aquaculture, Technical University of Denmark, North Sea Center, PO Box 101, 9850 Hirtshals, Denmark; 6 Department of Organismal Animal Physiology, Radboud University, Institute for Water and Wetland Research, Heyendaalseweg 135, 6525 AJ, Nijmegen, The Netherlands; 7 Department of Animal and Aquacultural Sciences, Norwegian University of Life Sciences, PO Box 5003, 1432 Ås, Norway

**Keywords:** stress, coping, serotonin, cortisol, neurochemistry

## Abstract

Signalling systems activated under stress are highly conserved, suggesting adaptive effects of their function. Pathologies arising from continued activation of such systems may represent a mismatch between evolutionary programming and current environments. Here, we use Atlantic salmon (*Salmo salar*) in aquaculture as a model to explore this stance of evolutionary-based medicine, for which empirical evidence has been lacking. Growth-stunted (GS) farmed fish were characterized by elevated brain serotonergic activation, increased cortisol production and behavioural inhibition. We make the novel observation that the serotonergic system in GS fish is unresponsive to additional stressors, yet a cortisol response is maintained. The inability of the serotonergic system to respond to additional stress, while a cortisol response is present, probably leads to both imbalance in energy metabolism and attenuated neural plasticity. Hence, we propose that serotonin-mediated behavioural inhibition may have evolved in vertebrates to minimize stress exposure in vulnerable individuals.

## 1. Background

The neurobiology of farmed fish is subject to expanding interest due to increasing concern for the welfare of production animals [1], and fishes are also progressively replacing mammalian models in biomedical research [2]. Neural signalling systems activated under stress are highly conserved [3–6], suggesting adaptive effects of their function. It is therefore a conundrum for evolutionary-based medicine that pathologies often arise from continued activation of the same systems [7]. One possibility is that pathologies may have evolved from behavioural responses that were adaptive in more primordial environments [8–11]. It is assumed that a mismatch between the historic and current environment leads to normally adaptive responses overriding self-correcting tendencies of emotional mechanisms, and this leads to pathologies [12]. Empirical investigation into this stance of evolutionary-based medicine is, however, scarce.

Here, we exploit a model in which such mismatch is prone to occur, namely, Atlantic salmon (*Salmo salar*) undergoing rapid domestication [13]. Under aquaculture conditions, it is common to find a proportion of farmed salmon, which are easily catchable at the surface and exhibit a small size, anorexia and a behaviourally inhibited profile. Such moribund fish, known as ‘drop outs’ or ‘loser fish’, are well known to farmers but very little is known about the aetiology leading up to this phenomenon [14,15]. We investigated serotonin (5-hydroxytryptamine, 5-HT) activity in the brain stem, which contains important monoaminergic nuclei innervating large parts of the brain [4], and plasma cortisol levels of surface-dwelling growth-stunted (GS) fish as well as healthy individuals occurring in a commercial aquaculture salmon farm. In the vertebrate brain, 5-HT-mediated signalling has a crucial role in energy regulation, neural plasticity, behavioural and emotional control, as well as neuroendocrine responses to stress [16,17]. Sustained serotonergic activation is associated with chronic stress and stress-induced pathologies, such as depression-like states, in several animal species [4,16,18–20]. We here report the novel observation that the serotonergic system in GS fish is not responsive to additional acute stress, while a significant corticosteroid response is maintained.

## 2. Material and methods

### 2.1. Experimental animals and facilities

**Figure 1.**
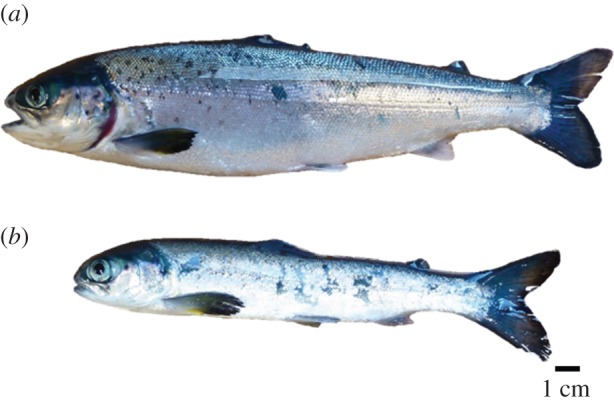
Representative pictures of healthy (*a*) and GS (*b*) fish from and aquaculture farm in the Langenuen Straight, Western Norway. (Photos: Ole Folkedal.)

GS and healthy fish (commercial strain, Aquagen AS) were selected during two sampling trials at a commercial salmon farm in the Langenuen Straight, Western Norway (60°N). Fish were sampled from two sea cages (25 × 25 × 30 m depth; approx. 18 750 m^3^) containing spring smolts. The fish were reared according to production standards, under a natural light regime and were fed formulated food given over two equally sized meals from 09.00 to 12.00 and 13.00 to 16.00. We sampled fish two (sampling 1; S1) and five (sampling 2; S2) months after seawater transfer to control for repeatability and production phase differences. In this study, we have categorized these two groups as: (i) *healthy*, normal schooling individuals with healthy behaviour and feeding responses, displaying high reactivity to environmental stimuli; and (ii) *GS fish*, which tended to position themselves towards the edge of the sea cage, close to the surface and without much reaction to environmental stimuli, including food pellets. Representative phenotypes are shown in figure 1, and number of individuals collected and their average length and weight are presented in table 1.

**Table 1.** Average length and weight (±s.d.) and number (*n*) of healthy and GS Atlantic salmon collected during first (S1) and second (S2) samplings at basal and acute stress conditions.

	unstressed		stressed	
	healthy	GS	healthy	GS
S1				
length (cm)	24.7 ± 0.6	18.9 ± 0.6	25.1 ± 0.7	17.8 ± 0.8
weight (g)	176 ± 13.1	50.3 ± 7.1	179 ± 16	42.4 ± 7.4
*n*	15	14	14	12
S2				
length (cm)	38.2 ± 1.2	25.4 ± 0.8	38.2 ± 1.1	26.1 ± 0.9
weight (g)	721 ± 81.4	145 ± 16.8	716 ± 54.9	162 ± 18.3
*n*	10	10	10	10

### 2.2. Acute stress treatment

All fish were captured by quickly netting them out of the cage with a dip net from 2 m depth close to the net wall. Healthy fish were lured towards the net wall by throwing out feed-pellets and were caught while feeding. Meanwhile, GS fish remained close to the net wall the whole time and were simply netted out of the cage. Fish from both groups were either sampled directly after capture or subjected to a 30 min acute stress test before sampling (see table 1 for *n*/group). Acute stress consisted of individual confinement in a circular 12 l plastic bucket containing seawater from a depth of 1 m (at approx. 12°C) for a period of 30 min after Pottinger & Carrick [21]. Oxygen levels were constantly monitored with an oxygen probe (Storvik HQ40, Storvik Aqua AS, Sunndalsøra, Norway) and kept above 6 mg l^−1^ to ensure that fish were not exposed to hypoxia [22].

### 2.3. Sampling protocol

Fish were euthanized with a strong overdose of 1 g l^−1^ MS-222 (Finquel®, Argent Chemical Laboratories, Redmond, WA, USA), which rendered them completely motionless (no opercular movement) within 10 s of immersion. Fish were rapidly weighed, fork length measured and a blood sample was taken from the caudal vessels with 23 G, 1 ml syringes containing the anticoagulant ethylene diamine tetra acetic acid (EDTA). Following centrifugation for 5 min at 9.289 rcf and 4°C, plasma samples were frozen and stored at −80°C for later analysis. Fish were decapitated and the brain stem was quickly excised within 2 min. Brain stems were wrapped in individually marked aluminium foil packets, snap-frozen in liquid nitrogen and stored at −80°C for later analysis of 5-HT neurochemistry.

### 2.4. Brain stem serotonergic neurochemistry

Frozen brain stems were homogenized in 4% (w/v) ice cold perchloric acid (PCA) containing 0.2% EDTA and 3,4-dihydroxybenzyl amine hydrobromide (DHBA, 40 ng ml^−1^) as an internal standard using a Potter–Elvehjem homogenizer. After spinning samples for 10 min at 15.493 rcf and 4°C, the supernatant was analysed by means of high-performance liquid chromatography (HPLC). The mobile phase was made up of 12 µM EDTA, 86 mM sodium phosphate and 1.4 mM sodium octyl sulfate in deionized water (resistance 18.2 MW), containing 7% acetonitrile set to pH 3.1 using phosphoric acid. The system contains a solvent delivery system (Shimadzu, LC-10AD), an auto-injector (Famos, Spark), a reverse-phase column (4.6 mm 100 mm, Hichrom, C18, 3.5 mm) and an ESA Coulochem II detector (ESA, Bedford, MA, USA) with two electrodes at −40 mV and +320 mV. A conditioning electrode with a potential of +40 mV was used to oxidize possible contaminants before analysis. Brain stem concentrations of 5-HT and the 5-HT catabolite 5-hydroxyindoleacetic acid (5-HIAA) were quantified by comparison with standards and corrected for recovery of the internal standard using HPLC software (CSW, Data Apex Ltd, the Czech Republic).

### 2.5. Cortisol radioimmunoassay

Plasma samples were diluted 2.2 times in assay buffer and cortisol assayed by radioimmunoassay (RIA) following the procedure described by Gorissen *et al*. [23]. Intra- and interassay variations were 12.5% and 3.5%, respectively, and cross-reactivity of the cortisol antibody (antibody [xm210]; Abcam, Cambridge, MA, USA) was as follows: cortisol 100%, 11-deoxycortisol 0.9%, prednisolone 5.6%, corticosterone 0.6%, 11-deoxycorticosterone, progesterone, 17-hydroxyprogesterone, testosterone, oestradiol and oestriol all less than 0.01%.

### 2.6. Statistical analyses

All datasets were tested by Levene's test for variance homogeneity, and values were either log- (cortisol, 5-HT and 5-HIAA) or arcsine-transformed (5-HIAA/5-HT ratios) when necessary. A two-way ANOVA was used to compare concentrations of 5-HT, 5-HIAA, cortisol and the 5-HIAA/5-HT ratios for each sampling, with fish type (GS versus healthy control) and treatment (basal versus acute stress) as independent variables, followed by a Tukey–Kramer honestly significant difference (HSD) post hoc test when a significant interaction effect was indicated.

## 3. Results

### 3.1. Brain stem 5-hydroxytryptamine neurochemistry

**Figure 2.**
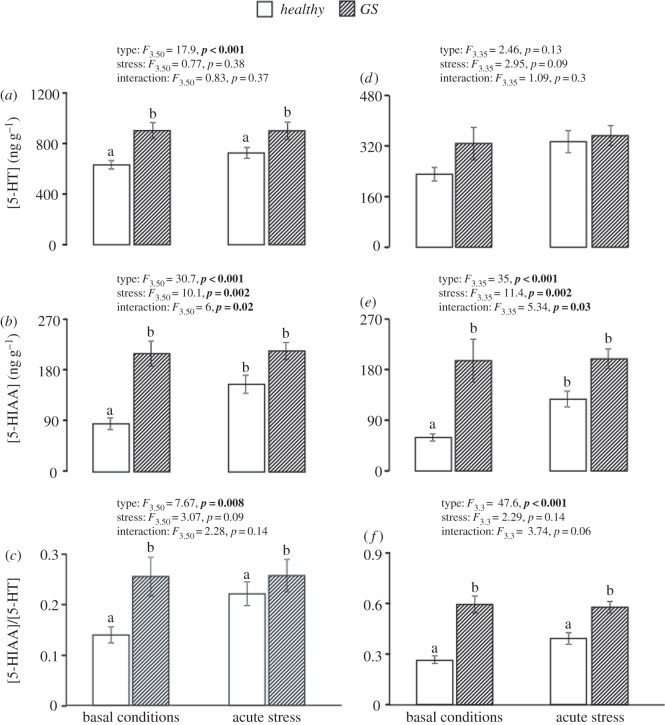
Effect of fish type (i.e. healthy versus GS) and stress (basal versus acute stress) on serotonin (5-HT) neurochemistry in the brain stem of Atlantic salmon at S1 (*a*–*c*) and S2 (*d*–*f*). Two-way ANOVA statistics are given in figure for each panel. Small letters indicate a fish-type effect or Tukey–Kramer HSD post hoc differences following a significant interaction effect. Data represent mean ± s.e.m.

We investigated 5-HT neurochemistry at basal conditions and after acute stress in groups of healthy and GS salmon at two different time points (two and five months following transfer to seawater rearing, i.e. S1 and S2, respectively). At both time points, GS fish sustained significantly higher levels of the principal 5-HT catabolite 5-HIAA compared with healthy controls (*p *< 0.001 in both samplings). Interestingly, whereas 5-HIAA levels were significantly increased by confinement stress in controls (post hoc *p*_S1_* *= 0.001, *p*_S2_* *= 0.002), 5-HIAA levels did not differ between basal and confinement stress (*p*_S1_* *= 0.96, *p*_S2_* *= 0.87) in the GS fish (figure 2*b*,*e*), indicating a blunted serotonergic response to stress in these individuals. Meanwhile, there was a significant effect of phenotype on the 5-HIAA/5-HT ratio (*p*_S1_* *= 0.008 and *p*_S2_ < 0.001) in both samplings, where healthy fish displayed lower ratios than GS fish (figure 2*c*,*f*). For 5-HT concentrations, there was a discrepancy between the two samplings, whereas there was a fish-type effect during S1 in which healthy fish had significantly lower 5-HT levels compared with GS fish (*p* < 0.01), this difference was not statistically significant in S2 (*p* = 0.13). Nevertheless, we found that on both samplings there was neither a stress nor an interaction effect on 5-HT (figure 2*a*,*d*).

### 3.2. Plasma cortisol levels

**Figure 3.**
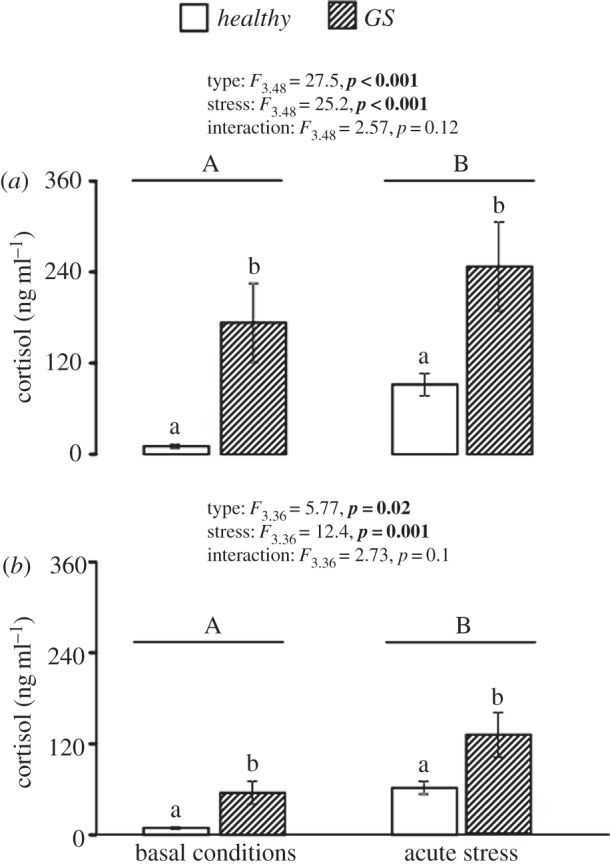
Effect of fish type (i.e. healthy versus GS) and stress (basal versus acute stress) on plasma cortisol concentrations in Atlantic salmon, at S1 (*a*) and S2 (*b*). Two-way ANOVA statistics are given in figure for each panel. Small and big letters indicate a type and treatment effect, respectively. Data represent mean ± s.e.m.

At both time points of sampling, plasma cortisol was affected by both type (*p*_S1_* *< 0.001 and *p*_S2_ = 0.02) and stress (*p* ≤ 0.001 in both samplings), with no interaction effect between the two variables (*p* = 0.1 in both). Plasma cortisol levels were significantly lower in healthy fish compared with GS fish. However, both groups responded with increased plasma cortisol levels to confinement stress (figure 3).

## 4. Discussion

The occurrence of GS fish is a common and costly occurrence in salmon aquaculture, affecting up to a quarter of the stock, but little has been done to elucidate the aetiology of this phenotype. We here present, for the first time, physiological data for GS fish, showing that elevated serotonergic activity is a main characteristic of the GS phenotype. That is, compared with healthy fish, GS salmon are characterized by increased basal brain serotonergic activity and cortisol production measured over two samplings several months apart, which suggest chronic activation of the serotonergic and hypothalamic-pituitary interrenal (HPI) axis systems [6,24,25]. Our findings support the view that research focusing on genetic markers for stress coping abilities in farmed fish is necessary to improve welfare and selection for breeding. Intriguingly, a growing body of evidence indicates that several depression-like syndromes are associated with increased serotonergic signalling (for a review, see [17]). In this context, it is interesting to note that the behavioural and serotonergic profile exhibited by GS fish is reminiscent of a depressed state, similar to those described in mammals [17,26–28]. However, further research is needed in order to corroborate this possibility.

Both 5-HIAA levels and the 5-HIAA/5-HT ratios as well as plasma cortisol levels observed during both samplings in GS fish at basal conditions indicate chronic serotonergic and HPI axis activation [6,24,25]. Notably, there were two discrepancies between both samplings. First, measured plasma cortisol and 5-HT levels were overall higher in all groups during S1, compared with S2. Both cortisol and 5-HT concentrations are known to vary with the life stage of the fish and most notably cortisol and 5-HT increase during smoltification (the metamorphosis that prepares salmon for the saltwater environment). That is, an increase during smoltification in brain 5-HT and plasma cortisol concentrations of up to 50% [29] and up to 100% [30], respectively, has been reported. In our experiment, fish were sampled shortly after smoltification (S1) and five months later (S2). In line with these reports, both 5-HT and plasma cortisol levels were higher at the sampling point closest to smoltification. Second, while basal serotonergic activity was found to be consistently higher, at both sampling points, in GS fish compared with healthy individuals, 5-HT concentrations were similarly higher in GS fish compared with healthy fish in S1 only. However, 5-HT concentrations do not directly reflect serotonergic signalling. Firstly, 5-HT is rapidly replaced by newly synthetized 5-HT intracellularly following its release at the synapse [31] and secondly, extracellular 5-HT concentrations reflect both 5-HT release and clearance from the synapse [17]. Instead, 5-HIAA levels and the 5-HIAA/5-HT ratio are used as a proxy for serotonergic activity [6,17,31,32], as the formation of the 5-HT catabolite 5-HIAA is almost exclusively a result of monoamine oxidase metabolizing 5-HT after release into the synapse and reuptake into the presynaptic neuron or surrounding cells. Our most important finding is that the serotonergic system of GS fish is unresponsive to acute novel stress. This phenomenon has, to the best of our knowledge, never been shown before, but is a classic example of allostatic overload in a physiological system (i.e. the inability of regulatory mechanisms to react to further challenge). The inability of the serotonergic system to respond to novel acute stressors, while a cortisol response is maintained, probably leads to both an imbalance in energy metabolism [17] and attenuated neural plasticity [5,33]. Therefore, the passive-behavioural phenotype characteristic of GS fish may be mediated by sustained serotonergic activation in order to minimize further stress exposure.

Although we have established that GS fish suffer from an elevated activation of the serotonergic system, the underlying causes of the increased serotonergic activity remains to be determined. There are multiple potential contributors to increased serotonergic activity in farmed salmon. For example, subordinate social rank within aquaculture populations is associated with chronically increased serotonergic activity [34]. Furthermore, the strains of Atlantic salmon used in aquaculture have gone through a rapid and intense domestication, and the aquaculture environment challenges individuals with a series of stressors that do not occur in nature [13]. For example, vaccinating fish shortly after smoltification (a common practice in aquaculture [35,36]) challenges homeostatic balance, induces autoimmunity [37] and decreases feed intake for several weeks [36]. Interestingly, both vaccination [38] and smoltification [29] alter serotonergic signalling and could lead to prolonged serotonergic activation. Thus, an environmental mismatch between natural (e.g. smoltification) and ‘artificial’ stressors (e.g. grading, vaccination) might leave some individuals more vulnerable to disease and behavioural syndromes [13]. Further investigations are, however, needed to pinpoint the proximate causes of the GS phenotype, e.g. the possible interaction between genetic effects, immune status and social hierarchies.

Future studies should focus on the environmental factors and genetic, behavioural and physiological characteristics underpinning GS fish, especially considering that individual differences in vulnerability are still unresolved [17,39]. We believe that these findings should encourage further exploration of both the evolutionary background and causative molecular mechanisms for 5-HT-induced behavioural inhibition. Such knowledge may be pivotal in advancing the development of new clinical approaches to stress-induced pathologies and may ultimately facilitate a shift in focus to the medical ideal of prevention rather than cure.

## Supplementary Material

Data file

## Data accessibility

All relevant data are within the paper and its electronic supplementary material.
